# Supplementary material for: The Novel Serine/Threonine Protein Kinase LmjF.22.0810 from Leishmania major May Be Involved in the Resistance to Drugs such as Paromomycin
Source: Biomolecules. 2019 Nov 11;9(11):723. doi: 10.3390/biom9110723 (PMC6920834; doi:10.3390/biom9110723)
Supplement: Supplementary file 1 [file biomolecules-09-00723-s001.pdf]

biomolecules

Supplemental Material

Supplemental Table S1. H-bonds found in LmJean3 kinase domain at least in 30% of the trajectory.

Donor	Acceptor	Occupancy (>30%)
HIS121-Side	ASP192-Side	84.22%
HIS127-Main	ASP192-Side	78.02%
LEU82-Main	VAL35-Main	70.98%
GLU9-Main	ARG26-Main	67.28%
ARG254-Side	SER248-Main	65.68%
SER236-Side	GLU103-Side	63.84%
ILE10-Main	ARG3-Main	63.49%
THR205-Side	TYR201-Main	62.99%
SER236-Main	GLU103-Side	62.69%
ARG51-Side	GLU47-Side	62.64%
ALA36-Main	LYS23-Main	62.24%
ARG24-Main	ASP12-Main	62.14%
TYR78-Main	VAL39-Main	61.94%
ARG128-Main	ASP192-Side	60.49%
HIS27-Main	CYS32-Main	59.69%
VAL114-Main	LEU110-Main	58.09%
ARG268-Side	GLU238-Side	56.44%
ARG58-Side	GLU70-Side	56.09%
PHE34-Main	VAL25-Main	55.29%
VAL25-Main	PHE34-Main	55.09%
SER195-Side	TYR176-Main	55.04%
VAL5-Main	TYR8-Main	54.40%
LYS37-Main	ILE80-Main	53.90%
ILE80-Main	LYS37-Main	53.70%
SER229-Main	PRO209-Main	52.70%
LEU72-Main	TYR79-Main	52.25%
TYR79-Main	LEU72-Main	52.15%
ILE38-Main	LYS21-Main	52.05%
HIS121-Main	VAL117-Main	51.40%
ARG118-Side	ASP258-Side	51.25%
ARG152-Side	ASP49-Side	51.05%
ASN64-Side	LEU143-Main	50.10%

Supplemental Table S1. H-bonds found in LmJean3 kinase domain at least in 30% of the trajectory.

Donor	Acceptor	Occupancy (>30%)
ARG26-Main	GLU9-Main	49.85%
LYS23-Main	ALA36-Main	49.75%
LEU137-Main	GLY88-Main	48.95%
ARG254-Side	ARG190-Main	47.45%
LYS41-Main	ASN76-Main	46.30%
ILE81-Main	GLU70-Main	45.75%
ARG268-Side	ASP264-Side	45.50%
HIS127-Side	SER146-Main	45.30%
VAL39-Main	TYR78-Main	44.41%
HIS27-Side	THR30-Side	42.81%
VAL22-Main	GLY15-Main	41.16%
ASN134-Side	ASP129-Main	40.61%
ARG190-Side	GLU252-Side	39.96%
LEU143-Main	GLN112-Side	38.76%
ILE199-Main	SER195-Main	37.91%
ILE69-Main	ILE81-Main	37.91%
VAL35-Main	LEU82-Main	37.36%
LEU110-Main	VAL106-Main	37.26%
ALA107-Main	GLU103-Main	36.76%
LYS41-Side	GLU47-Side	36.76%
VAL66-Main	ILE145-Main	36.51%
GLU103-Main	GLU103-Side	35.26%
GLN112-Side	ALA108-Main	35.01%
LYS21-Side	GLY17-Main	34.52%
ILE145-Main	ASN64-Main	33.37%
GLN67-Main	GLU83-Side	33.07%
SER146-Side	ASP147-Side	33.07%
LEU247-Main	CYS243-Main	32.72%
ILE111-Main	ALA107-Main	31.82%
LEU11-Main	ARG24-Main	31.82%
ILE28-Main	ASP7-Main	31.72%
VAL14-Main	VAL22-Main	30.22%

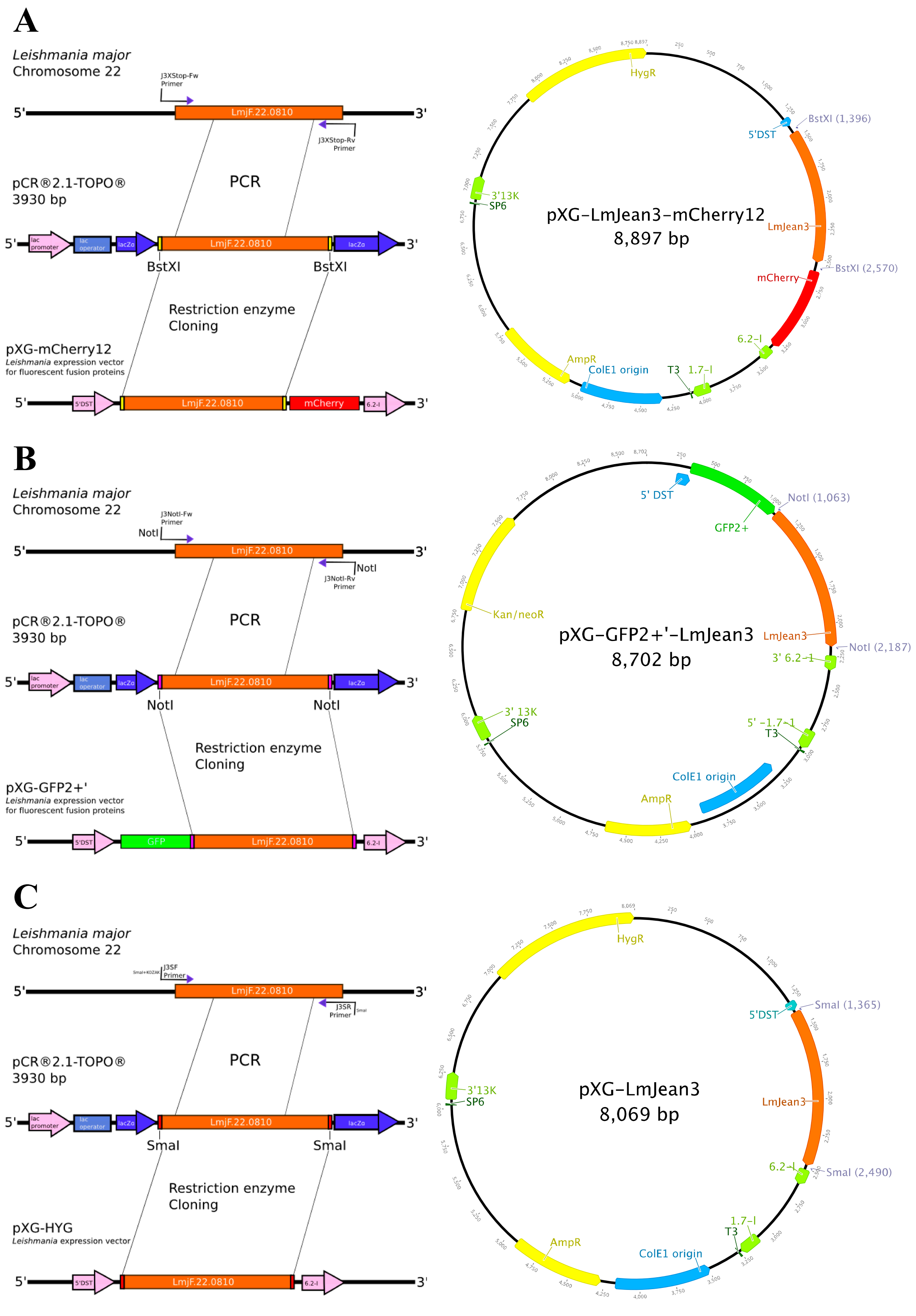

Supplemental Figure S1. Schematic representation of the cloning approach to generate the expression vectors: **A) pXG-*LmJean3*-mCherry12, B) pXG-GFP^{2+}-*LmJean3*,** and **C) pXG-Hyg-*LmJean3*.**

Supplemental Table S2. Primer sequences used for the construction of expression plasmids.

Primer	Sequence (5′→ 3′)
**J3NotI-Fw**	attgcggccgcATGCGGCGAGTCGGCGACTAC
**J3NotI-Rv**	ttgcggccgcCTAAACGTCTCCGCAGTATCC
**J3XStop-Fw**	acggtacaagtATGgGGCGAGTCGGCGACTAC
**J3XStop-Rv**	gaactagtAACGTCTCCGCAGTATCCACC
**J3SF**	aacccgggagtATGGGGCGAGTCGGCGACTAC
**J3SR**	tacccgggCTAAACGTCTCCGCAGTATCC

* Lower-case letters indicate added restriction enzyme sites.

Supplemental Table S3. Primer sequences used for qPCR analyses

Gene	Forward sequence (5′→ 3′)	Reverse sequence (5′→ 3′)
Jean3	AGCCGCCTCCACAGGGAAAG	GACGTACGCAATGCACCCCA
α-tubulin	ATGCGTGAGGCTATCTGCATCCACAT	TAGTGGCCACGAGCGTAGTTGTTCG
ABCH1	CGGGTTTGTCTTTCAGTCGT	CACCAGAGAGCATTGATGGA
ABCA3	ACGGGAACGGTAACATTGCT	GGCACAGCATCGAAATCGTC
ATPase 1-like	CTGTGCAACACAGTTCAGCC	TTGATGAGGCGATAGCCGAC
ABCC2	GCAGCCCCATGATGTTTATT	TCCGTTGCCTTCACTAGCTT
MRPA	CGCTTATCACCGACTGACGA	CCACCGCCTCCAAATCAGTA
GAPDH	ACCACCATCCACTCCTACA	CGTGCTCGGGATGATGTTTA

A

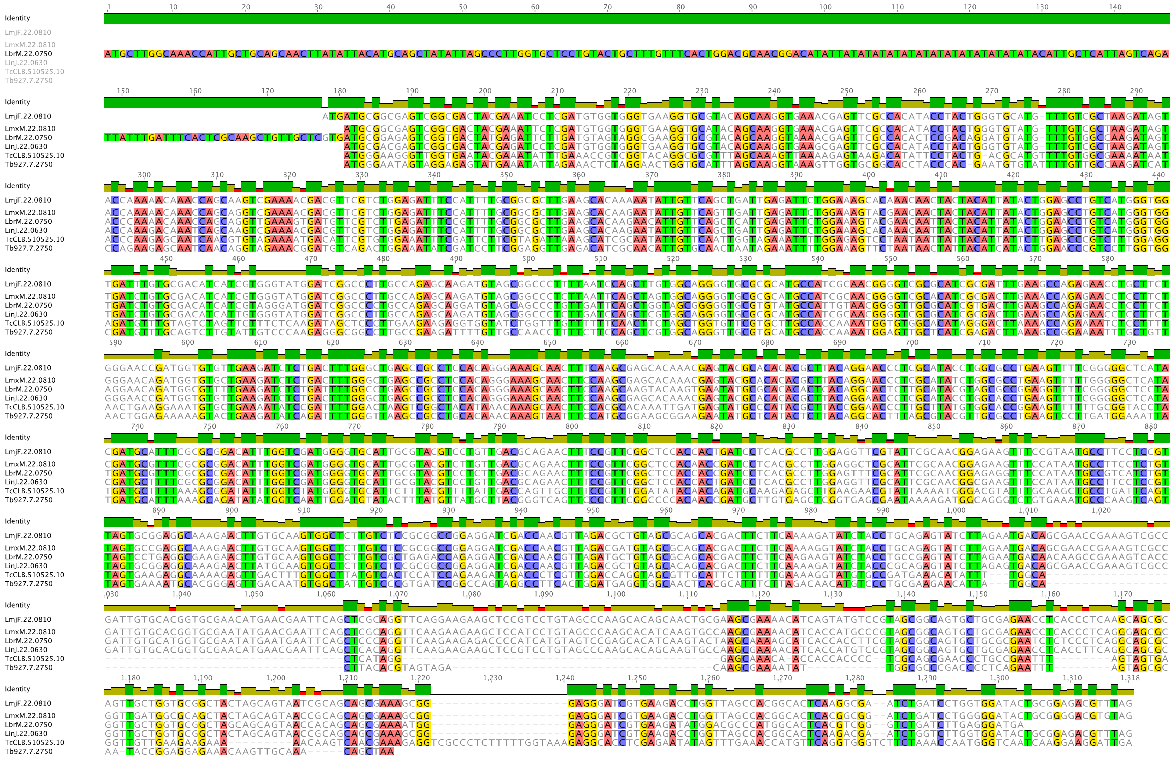

	LmjF.22.0810	LmxM.22.0810	LbrM.22.0750	LinJ.22.0630	TcCLB.510525.10	Tb927.7.2750
LmjF.22.0810		95.25	85.78	96.59	54.75	54.92
LmxM.22.0810	95.25		87.19	95.07	56.41	55.21
LbrM.22.0750	85.78	87.19		86.38	55.03	54.63
LinJ.22.0630	96.59	95.07	86.38		55.10	55.12
TcCLB.510525.10	54.75	56.41	55.03	55.10		66.49
Tb927.7.2750	54.92	55.21	54.63	55.12	66.49	

Identity

B

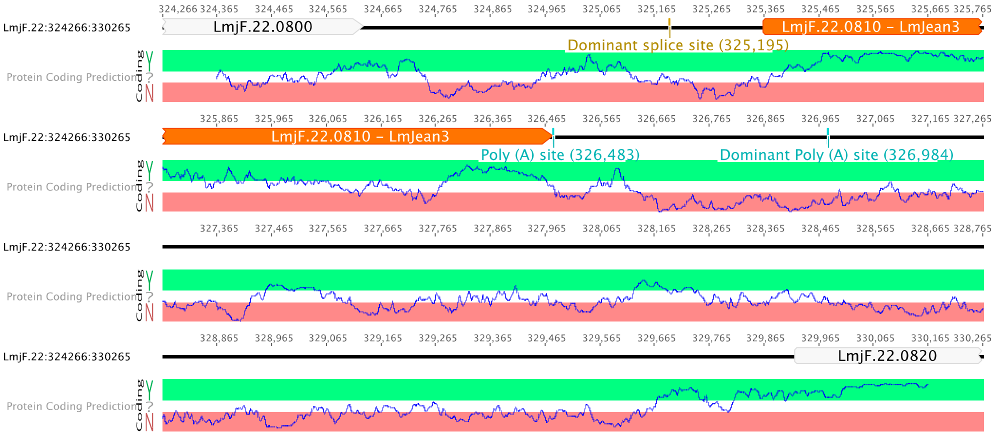

Supplemental Figure S2. ***LmjF.22.0810*** **orthologs are highly conserved. A**) *LmjF.22.0810* orthologs from *L. infantum*, *L. braziliensis*, *L. mexicana*, *T. brucei*, and *T. cruzi* sequence alignment. Agreements are highlighted. The table below the alignment indicates the percentage of conserved nucleotides found. **B**) Chromosomal surroundings of *LmjF.22.0810*. Top lane shows *L. major* chromosome 22 sequence positions. Splice site and Poly A sites are portrayed, as are both genes in the chromosome at both sides of *LmJean3* (*LmjF.22.0800* and *LmjF.22.0820*). The bottom line displays EMBOSS 6.5.7 protein coding prediction.

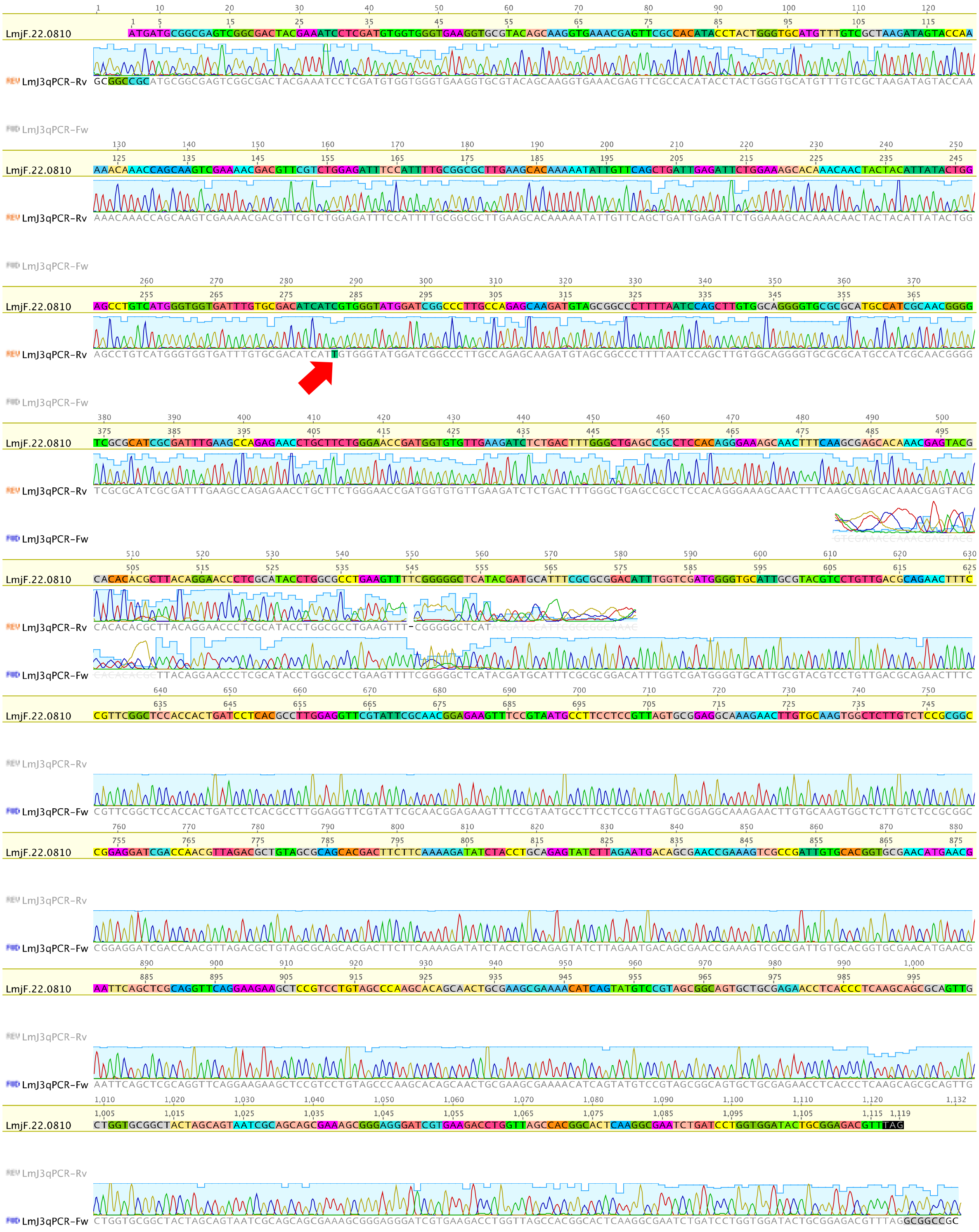

Supplemental Figure S3. LmJean3 sequencing study. Primers Jean3 (qPCR; **Supplemental Table S3**) were used to confirm *LmJean3* sequence from *L. major* (Lv39c5) parasites. Red arrow denotes the thymidine substitution found at position 282 (C282T).

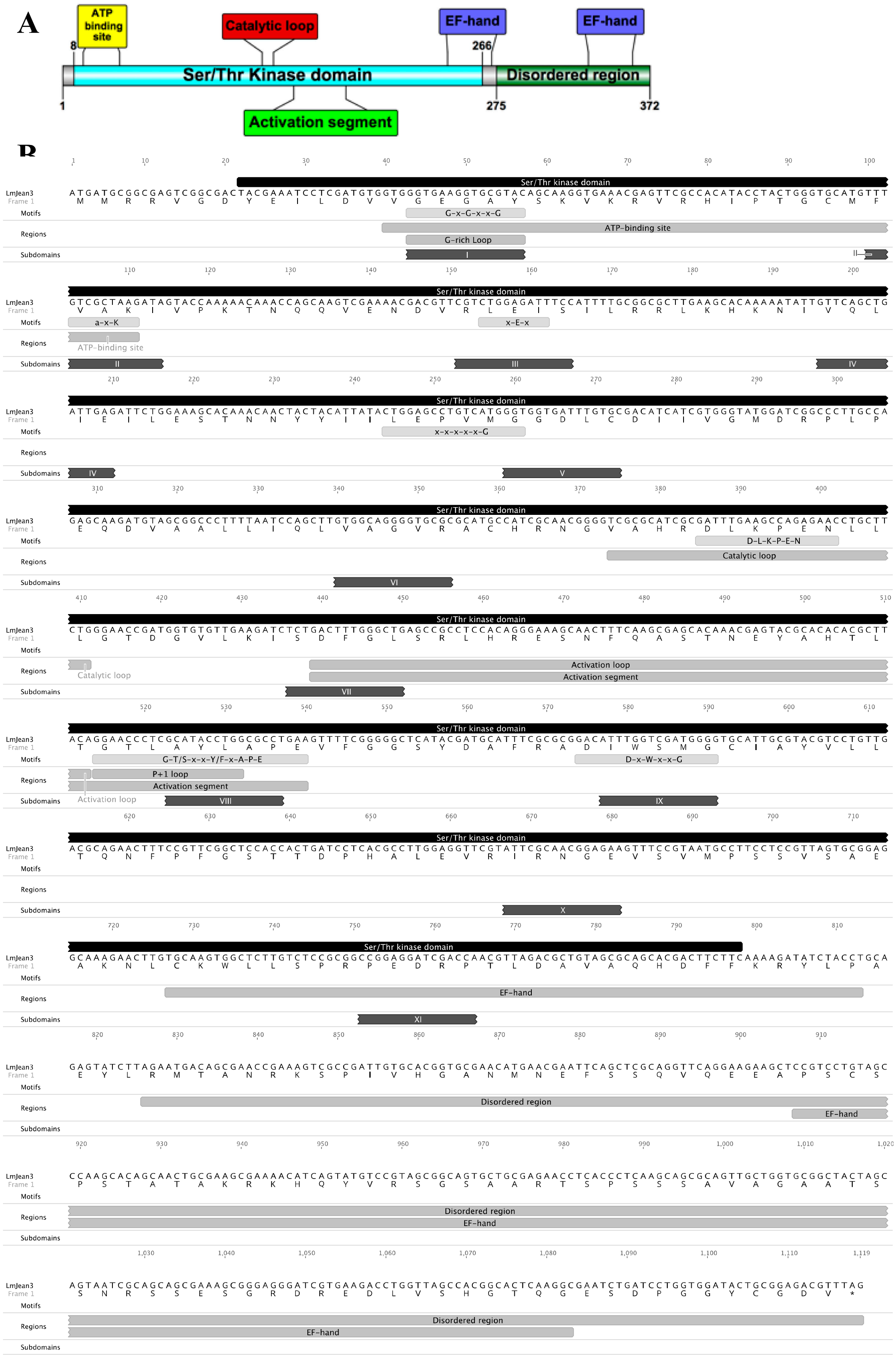

Supplemental Figure S4. ***LmjF.22.0810*** **encodes a putative Ser/Thr kinase and contains all the essential motifs and residues required for protein kinase activity among its 11 kinase subdomains.** *LmjF22.0810* sequence and the predicted protein translation product **(frame 1)** are shown. Each track displays the conserved motifs, regions, or subdomains of a catalytically active kinase. The invariant (gray boxes) and nearly invariant (gray squares) residues are annotated.

A

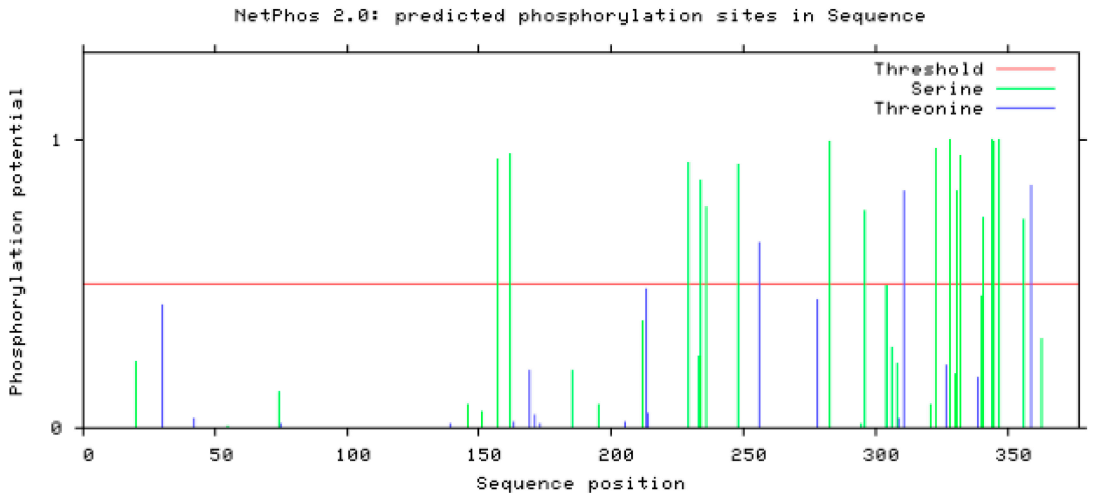

B

```
  372 Sequence
MMRRVGDYEILDVVGEGAYSKVKRVRHIPTGCMFVAKIVPKTNQQVENDVRLEISILRRLKHKNIVQLIEILESTNNYYI      80
ILEPVMGGDLCDIIVGMDRPLPEQDVAALLIQLVAGVRACHRNGVAHRDLKPENLLLGTDGVLKISDFGLSRLHRESNFQ     160
ASTNEYAHTLTGTLAYLAPEVFGGSYDAFRADIWSMGCIAYVLLTQNFPFGSTTDPHALEVRIRNGEVSVMPSSVSAEAK     240
NLCKWLLSPRPEDRPTLDAVAQHDFFKRYLPAEYLRMTANRKSPIVHGANMNEFSSQVQEEAPSCSPSTATAKRKHQYVR     320
SGSAARTSPSSSAVAGAATSSNRSSESGRDREDLVSHGTQGESDPGGYCGDV                                 400
................................................................................      80
............................................................................S...     160
.S..................................................................S....S.S....     240
.......S.......T..........................S............S..............T.........     320
..S....S..SS........S..SS.S........S..T.............                                 400

Phosphorylation sites predicted:        Ser: 17 Thr: 3
```

Supplemental Figure S5. LmJean3 phosphorylation sites prediction. A) The plot illustrates residue positions on the *X*-axis, while on the *Y*-axis the phosphorylation potential of each residue is drawn as green (serine) and blue (threonine) lines. Only serine and threonine residues that exceed the threshold (0.50) were considered phosphorylation sites. **B)** The 372 amino acids from the LmJean3 sequence are shown. Serine and threonine residues with a score greater than the threshold are plotted on the dotted line.

```
# WEBSEQUENCE Length: 426
# WEBSEQUENCE Number of predicted TMHs:  2
# WEBSEQUENCE Exp number of AAs in TMHs: 39.19509
# WEBSEQUENCE Exp number, first 60 AAs:  39.17402
# WEBSEQUENCE Total prob of N-in:        0.00087
# WEBSEQUENCE POSSIBLE N-term signal sequence
WEBSEQUENCE     TMHMM2.0        outside       1     9
WEBSEQUENCE     TMHMM2.0        TMhelix      10    27
WEBSEQUENCE     TMHMM2.0        inside       28    33
WEBSEQUENCE     TMHMM2.0        TMhelix      34    53
WEBSEQUENCE     TMHMM2.0        outside      54   426
```

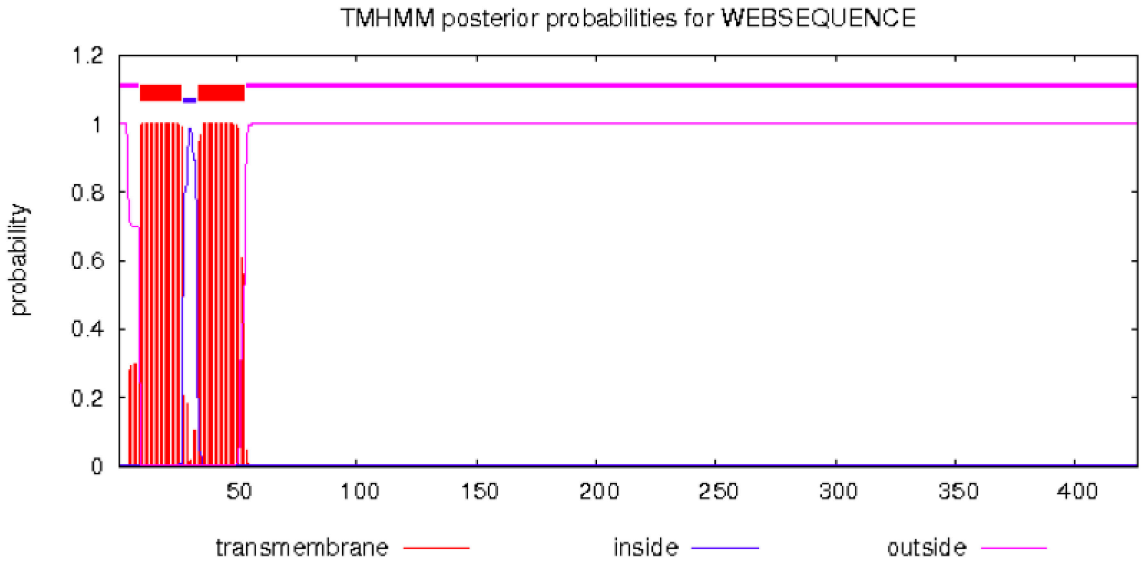

Supplemental Figure S6. ***L. braziliensis*** **Jean3 transmembrane helices prediction.** The prediction for the 426 amino acids from LbJean3 sequence is shown. The plot illustrates the predicted location of the intervening loop regions: The *X*-axis displays the residue positions, while the *Y*-axis shows the probability for each residue to be part of a membrane helix. The number of predicted transmembrane helices (TMH) was 2. The figure and the predictions were plotted by the TMHMM Server (http://www.cbs.dtu.dk/services/TMHMM/).

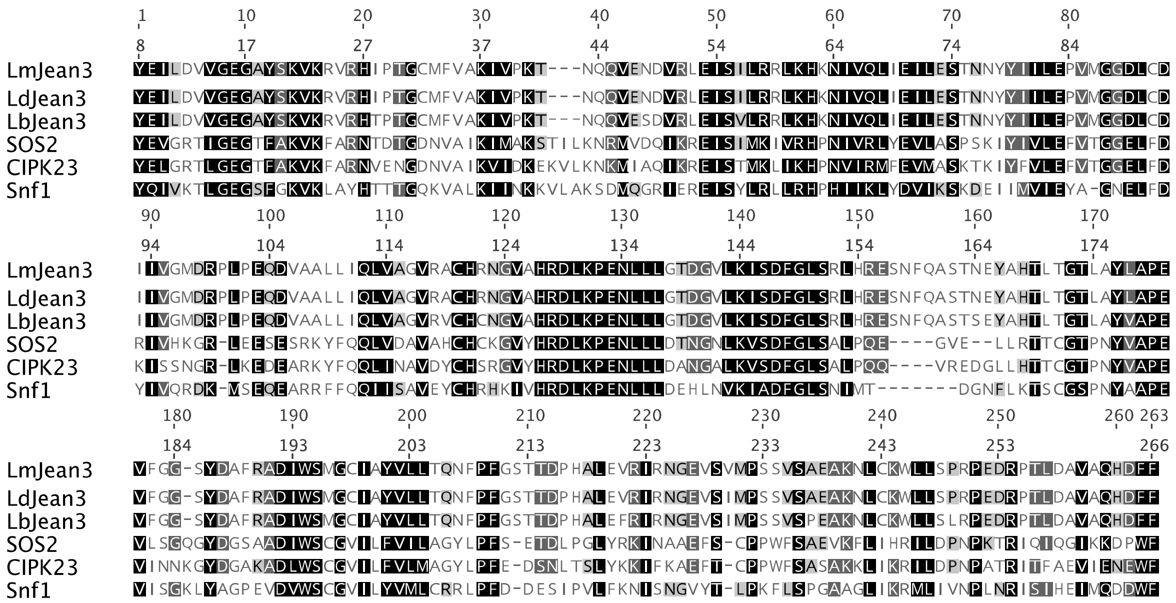

Supplemental Figure S7. Multiple sequence alignment of the kinase domain sequences from LmJean3, LdJean3, LbJean3, CIPK24/SOS22, and Snf1. Kinase domain sequences from *L. major Jean3* (XP_001683232), *L. donovani* Jean3 (XP_003860813), *L. braziliensis* Jean3 (XP_001564994), *A. thaliana* CIPK24/SOS2 (CCH26589), *A. thaliana* CIPK23 (NP_564353), and *S. cerevisiae* Snf1 (NP_010765). Residues are colored on the basis of their similarity under a BLOSUM62 score matrix [97]. Alignment was performed using the ClustalW iterative algorithm [53] implemented in Geneious v9.1.7 [36].

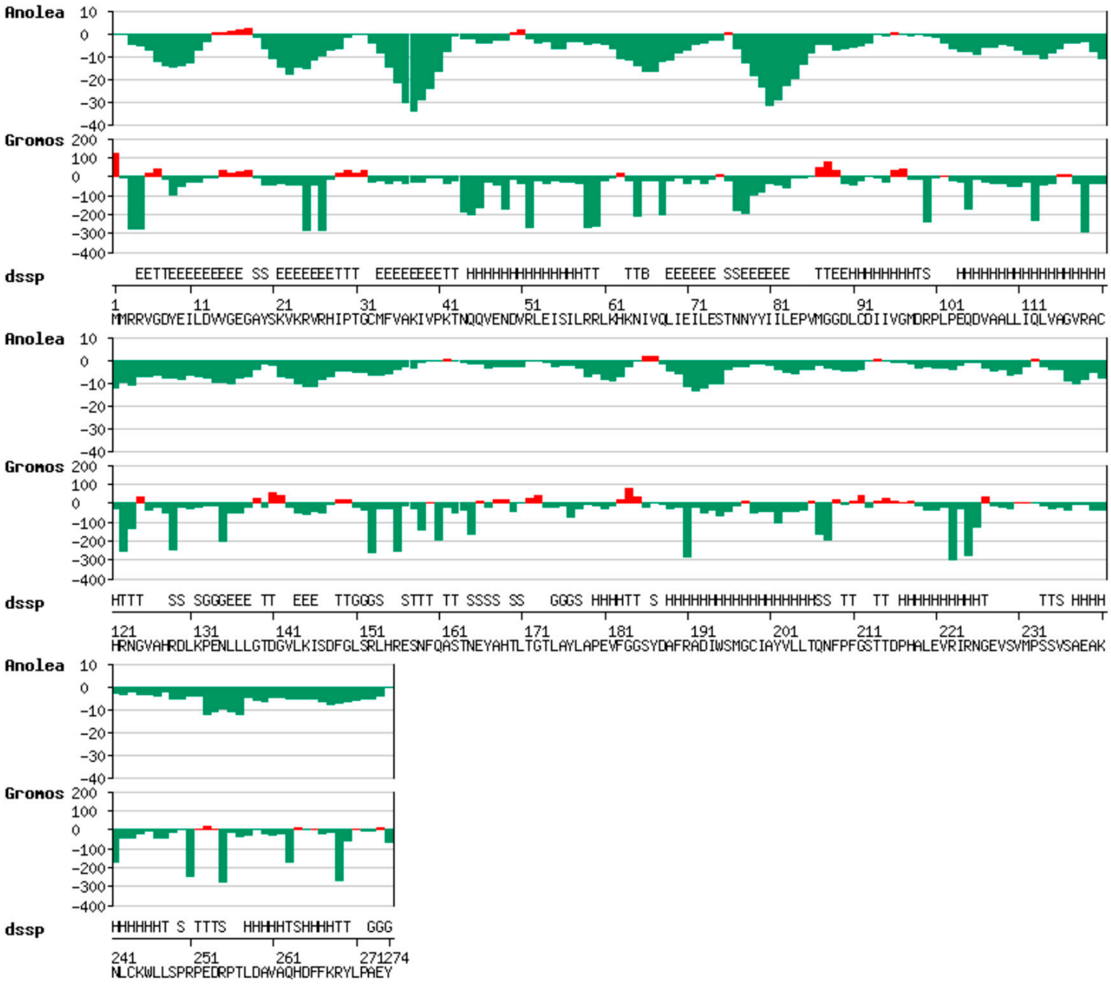

Supplemental Figure S8. ANOLEA and GROMOS quality estimation and DSSP defined secondary structure of LmJean3 model. The atomic empirical mean force potential (ANOLEA) was used to assess the packing quality of the models. The y-axis of the plot represents the energy of each amino acid of the protein chain. Negative energy values (in green) represent favorable energy environment, whereas positive values (in red) indicate unfavorable energy environment for a specific amino acid. GROMOS is a general-purpose molecular dynamics computer simulation package for the study of biomolecular systems and can be applied to the analysis of conformations obtained by experiment or by computer simulation. The y-axis of the plot represents the energy for each amino acid of the protein chain. Negative energy values (in green) depict a favorable energy environment, whereas positive values (in red) illustrate an unfavorable energy environment for a given amino acid. The DSSP program defines secondary structure, geometrical features, and solvent exposure of proteins, given atomic coordinates in Protein Data Bank (PDB) format. At the bottom, the protein sequence of LmJean3 is shown.

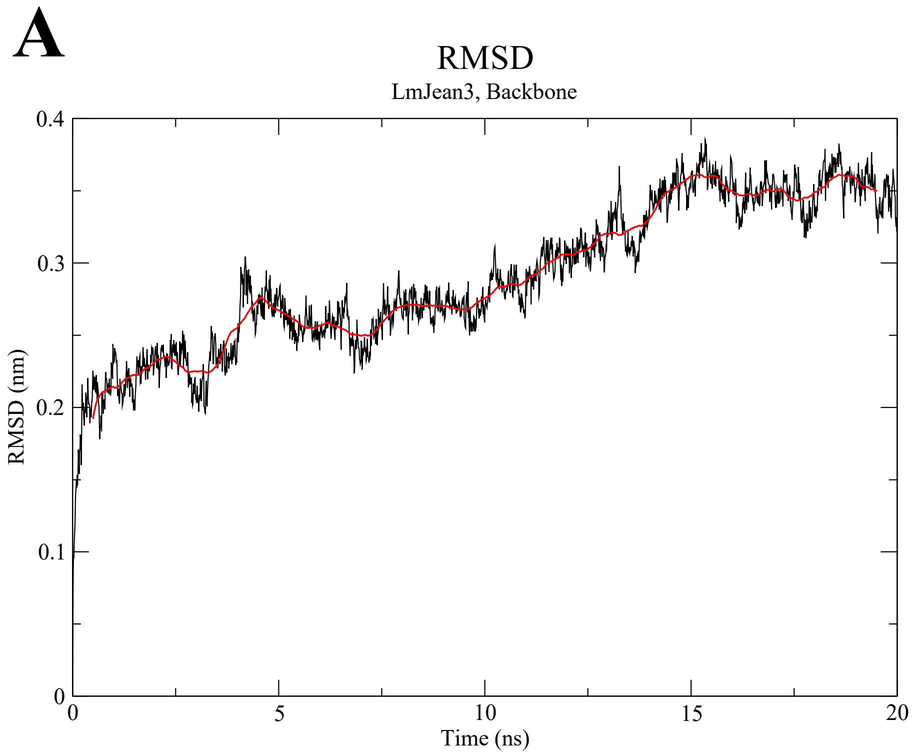

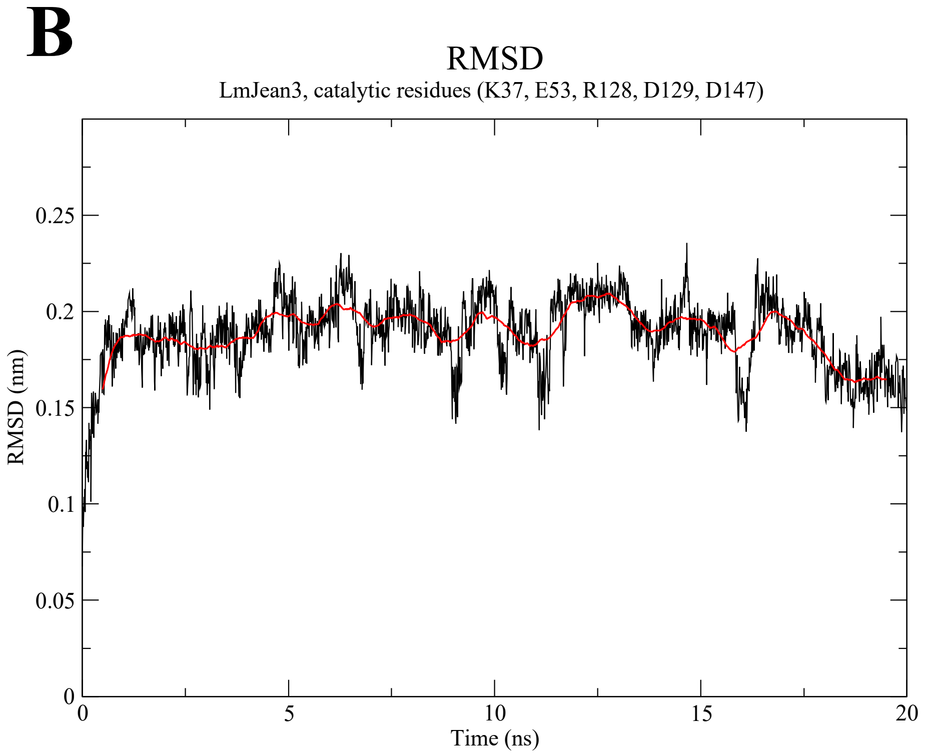

Supplemental Figure S9. Monitoring of MD trajectories. A) Time evolution of the RMSD of all protein Cα backbone atoms. **B)** Time evolution of the RMSD of the catalytically relevant residues from LmJean3 (K37, E53, R128, D129, and D147). The red line indicates the running averages with a length of 100 ps.

Supplemental Table S4. LmJean3 global quality estimation.

	**LmJean3**
**Modelled residues**	275
**RMSD (Å)**	1.87
**ERRAT (%)**	
Overall quality factor	98.11
**QMEAN6 (Z-score)**	
Overall Z-score	-0.42
Cβ interaction	-0.73
All-atom interaction	-0.33
Solvation	-0.43
Torsion	-0.09
Secondary structure agreement	0.47
Solvent accessibility agreement	-0.51
**SolvX**	
Overall score	-95.20
**Verify 3D (%)**	
3D–1D score ≥ 0.2	92.36
**ANOLEA (%)**	
High energy amino-acids	5.45
**Ramachandran plot (%)**	
Most favored regions	88.40
Additional allowed regions	10.80
Generously allowed regions	0.80
Disallowed regions	0

Supplemental Table S5. Predicted salt bridges in LmJean3 kinase domain. For each identified salt bridge, the average center of mass (COM) distance between each residue, the number of frames, and their persistence during the trajectory were calculated.

Donor	Acceptor	COM distance (<4.0 Å)	Frames	Occupancy (%)
LYS131	ASP129	2.80 ± 0.23	1970	98.45
LYS37	ASP147	3.03 ± 0.52	1650	82.46
LYS144	GLU83	3.11 ± 0.32	1941	97.01
ARG254	GLU180	3.30 ± 0.07	2002	100.00
LYS21	GLU16	3.41 ± 0.78	1756	87.71
ARG152	GLU53	3.53 ± 0.44	1715	85.66
ARG250	ASP253	3.62 ± 0.88	1886	94.21
LYS41	GLU73	3.65 ± 0.37	1931	96.45
ARG224	GLU220	3.70 ± 0.46	1999	99.85
ARG4	GLU9	3.93 ± 0.85	1711	85.46

biomolecules

MDPI

Supplemental Table S6. LmJean3 SiteMap property values and Dscore[a].

Site	Dscore	SiteScore	Size (site points)	Volume (Å^3)	exposure	enclosure	contact	phobic[b]	philic[b]	balance[b]	don/acc[c]
A	1.025	1.045	184	502.84	0.575	0.765	0.982	0.464	1.145	0.405	0.779
B	0.848	0.916	77	228.09	0.609	0.707	0.969	0.258	1.230	0.210	0.739
C	0.791	0.795	57	100.50	0.676	0.613	0.815	0.772	0.878	0.879	0.602
D	0.629	0.750	48	76.49	0.484	0.660	0.899	0.295	1.282	0.230	0.423
E	0.587	0.620	30	99.13	0.746	0.560	0.696	0.500	0.811	0.617	0.609

[a]Druggability score.

[b]These properties, labeled "phob" and "phil", measure the relative hydrophobic and hydrophilic character of the site. The "balance" property expresses the ratio of the two.

[c]Indicates the degree to which a well-structured ligand might be expected to donate, rather than accept, hydrogen bonds.

Supplemental Table S7. LmJean3 site B docking results*.

Rank	ZINC ID	Chemical Name	Chemical Structure	DockScore	Penalties	HBPenal[a]	ExposPenal[b]	RotPenal[c]	EpikStatePenalty[d]
1	60183170	Paromomycin	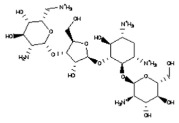	-11.46	0.00	0.00	0.17	0.13	0.00
2	71928289	Neomycin stereoisomer A	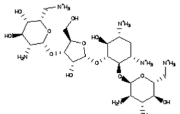	-10.93	0.00	0.00	0.10	0.11	0.00
3	04096846	Rutin	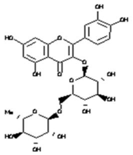	-10.84	0.00	0.00	0.04	0.09	0.00
4	60183167	Paromomycin stereoisomer	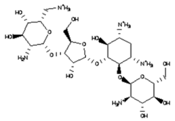	-10.66	0.00	0.00	0.19	0.13	0.00
5	71928290	Neomycin stereoisomer B	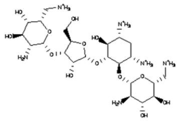	-10.24	0.00	0.00	0.79	0.08	0.00

*Chemical compounds are ranked according to their docking score. Penalties for each ligand are included in the table.
[a]Penalty for ligands with large hydrophobic contacts and low H-bond scores.
[b]Penalty for solvent-exposed ligand groups; cancels van der Waals terms.
[c]Rotatable bond penalty.
[d]Epik state penalties for ionization or tautomeric states that dock in preference to the most common state at physiological pH.

0

Supplemental Table S7. LmJean3 site B docking results*.

Rank	ZINC ID	Chemical Name	Chemical Structure	DockScore	Penalties	HBPenal[a]	ExposPenal[b]	RotPenal[c]	EpikStatePenalty[d]
6	03977803	Diosmin stereoisomer	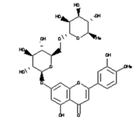	-9.66	0.00	0.00	0.08	0.09	0.00
7	71928292	Neomycin stereoisomer C	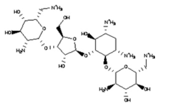	-9.58	0.00	0.00	0.39	0.11	0.00
8	03794794	Mitoxantrone	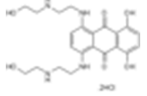	-9.14	0.00	0.00	0.20	0.52	0.00
9	08214692	Tobramycin	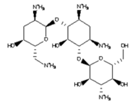	-9.06	0.00	0.00	0.02	0.19	0.00
10	04098512	Diosmin	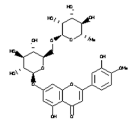	-9.05	0.00	0.00	0.00	0.09	0.00
11	33359835	Amikacin stereoisomer	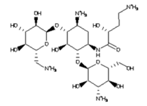	-9.04	3.00	0.00	0.05	0.19	0.00

*Chemical compounds are ranked according to their docking score. Penalties for each ligand are included in the table.
[a]Penalty for ligands with large hydrophobic contacts and low H-bond scores.
[b]Penalty for solvent-exposed ligand groups; cancels van der Waals terms.
[c]Rotatable bond penalty.
[d]Epik state penalties for ionization or tautomeric states that dock in preference to the most common state at physiological pH.

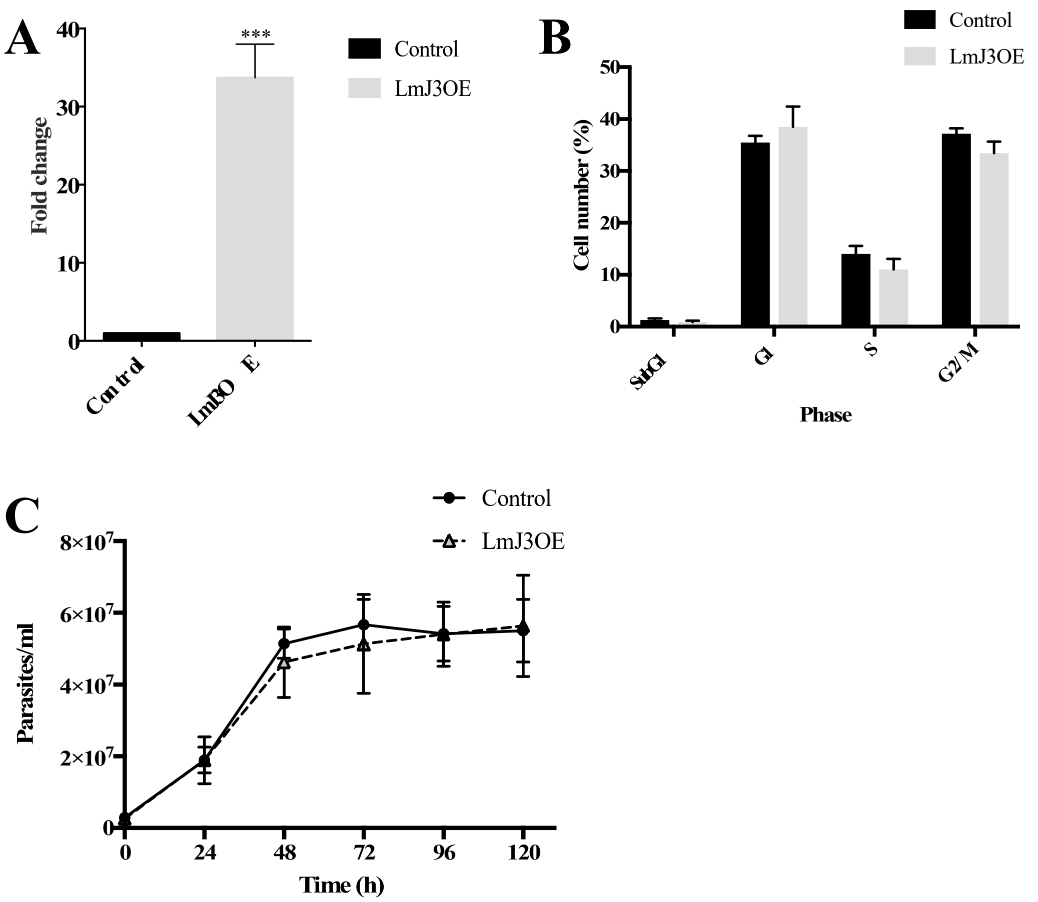

Supplemental Figure S10. Generation of *LmJean3*-overexpressing parasites. **A**) The rate of overexpression of *Jean3* was measured by qPCR in the control (pXG-*Hyg*) and *LmJean3*-overexpressing (LmJ3OE; pXG-*LmJean3*) strains. **B)** The cell cycle distribution of control and LmJ3OE parasites was evaluated by FACS analysis. The percentage of cells found in each phase of the cell cycle was similar between the control and LmJ3OE samples. **C)** The growth of control and LmJ3OE parasites was evaluated for 120 h. The growth curves of the two strains displayed analogous shape and distribution. Data are represented as the means (± SD) from three independent experiments (** P<0.01).

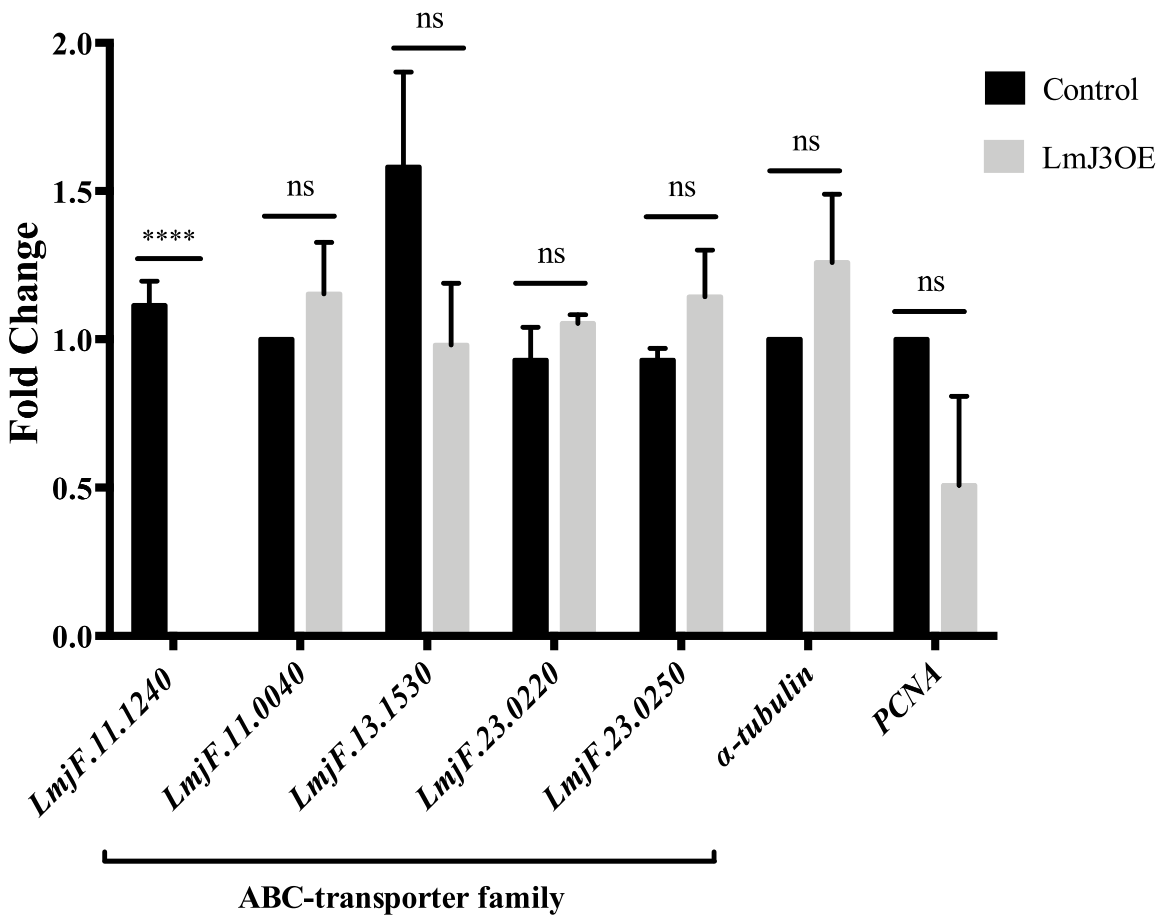

Supplemental Figure S11. **Gene expression analysis in LmJ3OE and control parasites.** The expression of genes of the ABC-transporter family (*LmjF.11.1240, LmjF.11.0040, LmjF.13.1530, LmjF.23.0220, LmjF.23.0250*), *α-tubulin*, and *PCNA* were quantified by qPCR. Bars represent the means (± SEM) from three independent experiments (ns, non-significant, *** P<0.001).

biomolecules

Supplemental Table S8. Drug activity profile of *L. major* cell lines. Promastigotes were grown as described in *Materials and Methods* for 48 and 72 h at 26 °C in the presence of increasing drug concentrations. Half-maximal effective concentrations (EC_{50}) were measured using an MTT-based assay. Results are expressed as means [± standard deviation (SD)] from three independent experiments.

**Compound**	**EC_{50} (µM) mean ± SD**			
	pXG-*Hyg*		pXG-*LmJean3*	
	48h	72h	48h	72h
**Paromomycin**	67.57 ± 8.49	120.00 ± 9.45	224.00 ± 44.85 (**)	210.60 ± 11.60 (***)
**Geneticin**	2.37 ± 0.41	2.55 ± 0.53	4.87 ± 0.29 (***)	5.24 ± 0.63 (**)
**Amphotericin B**	0.09 ± 0.02	0.16 ± 0.02	0.05 ± 0.01 (*)	0.10 ± 0.004 (*)
**Miltefosine**	8.57 ± 1.11	8.82 ± 0.70	5.97 ± 0.38 (*)	5.83 ± 1.39 (*)

p values <0.05 were considered statistically significant (*$p < 0.05$, ** $p < 0.01$, *** $p < 0.001$).
